# Phosphodiesterase 10A Upregulation Contributes to Pulmonary Vascular Remodeling

**DOI:** 10.1371/journal.pone.0018136

**Published:** 2011-04-11

**Authors:** Xia Tian, Christina Vroom, Hossein Ardeschir Ghofrani, Norbert Weissmann, Ewa Bieniek, Friedrich Grimminger, Werner Seeger, Ralph Theo Schermuly, Soni Savai Pullamsetti

**Affiliations:** 1 Medical Clinic II/V, University Hospital, Giessen, Germany; 2 Max-Planck-Institute for Heart and Lung Research, Bad Nauheim, Germany; Vanderbilt University Medical Center, United States of America

## Abstract

Phosphodiesterases (PDEs) modulate the cellular proliferation involved in the pathophysiology of pulmonary hypertension (PH) by hydrolyzing cAMP and cGMP. The present study was designed to determine whether any of the recently identified PDEs (PDE7-PDE11) contribute to progressive pulmonary vascular remodeling in PH. All *in vitro* experiments were performed with lung tissue or pulmonary arterial smooth muscle cells (PASMCs) obtained from control rats or monocrotaline (MCT)-induced pulmonary hypertensive (MCT-PH) rats, and we examined the effects of the PDE10 inhibitor papaverine (Pap) and specific small interfering RNA (siRNA). In addition, papaverine was administrated to MCT-induced PH rats from day 21 to day 35 by continuous intravenous infusion to examine the *in vivo* effects of PDE10A inhibition. We found that PDE10A was predominantly present in the lung vasculature, and the mRNA, protein, and activity levels of PDE10A were all significantly increased in MCT PASMCs compared with control PASMCs. Papaverine and PDE10A siRNA induced an accumulation of intracellular cAMP, activated cAMP response element binding protein and attenuated PASMC proliferation. Intravenous infusion of papaverine in MCT-PH rats resulted in a 40%–50% attenuation of the effects on pulmonary hypertensive hemodynamic parameters and pulmonary vascular remodeling. The present study is the first to demonstrate a central role of PDE10A in progressive pulmonary vascular remodeling, and the results suggest a novel therapeutic approach for the treatment of PH.

## Introduction

Pulmonary arterial hypertension (PAH) is a fatal disease characterized by progressively elevated pulmonary vascular resistance, which results from vasoconstriction, vascular remodeling and *in situ* thrombosis. These events lead to right ventricular hypertrophy and right heart failure [1]. All cell types of the vessel wall, including pulmonary arterial smooth muscle cells (PASMCs), endothelial cells and adventitial fibroblasts, are involved in this remodeling process [2]. Although the underlying mechanisms of pulmonary vascular remodeling in PAH are not completely understood, therapies targeting reduced prostacyclin synthesis, increased endothelin signaling and increased cyclic nucleotide phosphodiesterase (PDE) levels have been approved for the treatment of PAH [3]–[5].

Phosphodiesterases comprise a family of 11 isoforms (PDE1-PDE11) that each have different capacities for hydrolyzing cAMP, cGMP, or both. Because cAMP and cGMP are ubiquitous second messengers, PDEs are involved in many important signaling pathways that regulate proliferation, migration, and differentiation [6], [7]. Current evidence suggests that individual isozymes modulate distinct regulatory pathways in the cell, which are mainly determined by their sub-cellular localization [7]. PDE1A has been reported to translocate to the nucleus in synthetic proliferating vascular smooth muscle cells (SMCs) [8]. In addition, sub-isoforms of PDE4 have been shown to have diverse functions in subcellular pools of cAMP that result from compartmentalization [9].

Interestingly, the expression and activities of PDEs have been reported to be altered in both experimental and human PAH [10]. Expression profiling of single members of the PDE superfamily in healthy and remodeled pulmonary vasculature revealed that the PDE1, PDE3 and PDE5 isoforms are differentially regulated [11]–[13]. In preclinical and clinical studies, we have shown that the inhibition of PDE1 by 8-methoxymethyl-IBMX (8MM-IBMX) [11] and PDE5 by sildenafil [4], [12] stabilizes second messenger signaling and regulates vascular remodeling, vascular tone and optimization of gas exchange. Moreover, in monocrotaline (MCT)-induced PH (MCT-PH) rats, inhibition of PDE3 and PDE4 has been shown to partly reverse the pathological inward remodeling of PAH [14], [15].

The roles of the recently identified PDEs (PDE7-PDE11) in PAH are complicated and not well understood. Among them, PDE7 and PDE8 are cAMP-specific, PDE9 is cGMP-specific, and PDE10A and PDE11 are dual-substrate PDEs [16]. The cellular- and subcellular-specific distribution and substrate specificity of these newly identified PDEs may provide important insights into the pathology and pathophysiology of PAH.

The aim of the present study was to characterize the expression pattern of newly identified PDEs (PDE7-PDE11) in lung tissue and primary PASMCs from control and MCT-PH rats to identify potential therapeutic targets in the PDE family that are involved in the pathogenesis of PAH. Because the results showed a significant increase of PDE10A in the pulmonary hypertensive vasculature, we addressed the specific contribution of PDE10A to the vascular remodeling in PAH by employing small interfering RNA (siRNA) or an inhibitor in our *in vitro* and *in vivo* studies.

## Methods

### Patients

Human lung tissue was obtained from 4 donors and 4 patients with idiopathic PAH (IPAH) who underwent lung transplantation. The study protocol for human tissue donation was approved by the ethics committee (Ethik Kommission am Fachbereich Humanmedizin der Justus Liebig Universität Giessen) of the University Hospital Giessen (Giessen, Germany) in accordance with national law and “Good Clinical Practice/International Conference on Harmonisation” guidelines (AZ 31/93). Written informed consent was obtained from each individual patient or the patient's next of kin.

### Animals

Adult male Sprague Dawley rats (250–300 g in body weight; Charles River Laboratories, Germany) were randomized for a subcutaneous injection of saline or 60 mg/kg MCT (Sigma-Aldrich) to induce pulmonary hypertension (PH) [17]. Both the University Animal Care Committee and the Federal Authorities for Animal Research of the Regierungspräsidium Giessen (Hessen, Germany) approved the study protocols (AZ GI 20/10 Nr. 52/2009).

### Surgical preparation and hemodynamic measurement

The animals were classified into the following three groups: 1) rats injected with saline (Control, n = 9); 2) MCT-injected rats subjected to mini-pump implantation from day 21 to day 35 with saline (MCT [35 d], n = 8) or 3) MCT-injected rats subjected to mini-pump implantation from day 21 to day 35 with 1 µg/kg/min papaverine (Sigma-Aldrich) (MCT [35 d]/papaverine, n = 8). 3 weeks after MCT injection, rats were subjected to treatment for 2 weeks by implantation of osmotic mini-pumps (Alzet Model 2ML2, Durect). On day 21, after the rats were anesthetized, a mini-pump filled with 2 ml of saline or papaverine (5 mg/ml) was implanted in the dorsal subcutaneous region under sterile conditions, and a tunneled catheter (PE 50 tubing) was inserted into the left jugular vein. After wounds were closed with sutures, the rats were recovered from anesthesia by an intraperitoneal injection of naloxone and atipazemole (50 and 100 µg/kg, respectively). At the end of the treatment, the rats were anesthetized with an intraperitoneal injection of ketamine (9 mg/kg body mass) and medetomidine (100 µg/kg body mass), which was followed by an intramuscular injection of heparin (50 IU/kg body mass) to measure the hemodynamic parameters. The rats were then tracheotomized and ventilated at a frequency of 60 breaths/min with positive end expiratory pressure at 1 cm H_2_O. To measure right ventricular pressure, a right heart PE 50 catheter was inserted through the right jugular vein, and a polyethylene catheter was inserted into the left carotid artery to measure arterial pressure. After measurements, the left lung was fixed for histology in 3% paraformaldehyde solution, and the right lung was snap frozen in liquid nitrogen after exsanguinations [18].

### Histological assessment of the degree of muscularization of small pulmonary arteries

Three-µm lung sections from blocks fixed in 3% paraformaldehyde solution were used for double staining with anti-von Willebrand factor antibody (1∶900 dilution, Dako) and anti α-smooth muscle actin antibody (1∶900 dilution, Sigma-Aldrich) to analyze small peripheral pulmonary artery muscularization. In each rat, 80 to 100 intra-acinar arteries (20–50 µm) were categorized as fully muscularized, partially muscularized, or nonmuscularized, as previously described [18].

### Isolation of pulmonary arterial smooth muscle cells (PASMCs)

Rat PASMCs were cultured from peripheral small pulmonary artery explants as previously described [19]. Small pulmonary arteries were freshly obtained from rats and maintained in Hank's balanced salt solution (HBSS, Gibco) supplemented with penicillin (100 units/ml, PAN) and streptomycin (100 units/ml, PAN). Under a dissecting microscope, the adventitia layer was removed by microdissection. Arterial segments were cut open along the longitudinal axis, and the endothelium was gently removed by scraping the luminal surface. The arteries were minced into 1 mm^2^ explant pieces and maintained in Dulbecco's modified Eagle's medium/F12 (DMEM/F12, Gibco) supplemented with 10% fetal bovine serum (FBS, Biowest), penicillin (100 units/ml), streptomycin (100 units/ml), and 2 mM L-glutamine (PAN). After 5 days, PASMCs started to migrate from the explants, and this was followed by 10 days of subculturing. Early-passage (passages 2–5) PASMCs were used for all experiments. Cells were positively stained for α-smooth muscle actin by immunocytochemistry, and the expression of vascular smooth muscle cell (VSMC) phenotypic genes (α-smooth muscle actin, smooth muscle-myosin heavy chain, and calponin) was confirmed by polymerase chain reaction (PCR) (data not shown). Every experiment was performed with primary PASMCs isolated from at least 3 individual rats.

### RNA isolation and cDNA synthesis

For cDNA synthesis, total RNA from tissues or cells was extracted using Trizol® (Invitrogen) according to the manufacturer's instructions. One µm of RNA was used for reverse transcriptase polymerase chain reaction (RT-PCR) in a total volume of 20 µl with oligo(dT)_15_ primer using the ImProm-II reverse transcription system (Promega) according to the manufacturer's instructions.

### Quantitative real-time polymerase chain reaction (qRT-PCR)

The intron-spanning primer pairs (Metabion) were designed using the Primer3 program, and these primer pairs are shown in Table 1. qRT-PCR was performed on an Mx3000P® QPCR System machine (Stratagene) using SYBR® GreenER™ qPCR SuperMix Universal kits (Invitrogen). Using the MxPro™ QPCR software, a dissociation curve was generated for each gene to ensure single product amplification, and we determined the threshold cycle (Ct value) for each gene. The comparative 2^−ΔΔCt^ method was used to analyze mRNA fold changes between control and MCT group, which was calculated as ratio = 2^−(ΔCt control-ΔCt MCT)^, where Ct is the cycle threshold and ΔCt (Ct target - Ct reference) is the Ct value normalized to the reference gene porphobilinogen deaminase (PBGD) obtained for the same cDNA samples. Each reaction was run in duplicate and repeated in three independent experiments. The calculated 2^−ΔΔCt^ was transformed into a percentage using the control value as 100% to indicate the mRNA expression.

**Table 1:** Primer pairs of rat PDEs for quantitative realtime-PCR.

Gene	Forward Primer	Reverse Primer
**PDE1A**	5′- ATCAGCCACCCAGCCAAA -3′	5′- GGAGAAAACGGAAGCCCTAA -3′
**PDE3A**	5′- CACAAGCCCAGAGTGAACC -3′	5′- TGGAGGCAAACTTCTTCTCAG -3′
**PDE3B**	5′- GTCGTTGCCTTGTATTTCTCG -3′	5′- AACTCCATTTCCACCTCCAGA -3′
**PDE4A**	5′- CGACAAGCACACAGCCTCT -3′	5′- CTCCCACAATGGATGAACAAT -3′
**PDE7A**	5′- GAAGAGGTTCCCACCCGTA -3′	5′- CTGATGTTTCTGGCGGAGA -3′
**PDE7B**	5′- GGCTCCTTGCTCATTTGC -3′	5′- GGAACTCATTCTGTCTGTTGATG-3′
**PDE8A**	5′- TGGCAGCAATAAGGTTGAGA -3′	5′- GAATGTTTCCTCCTGTCTTT -3′
**PDE8B**	5′- TCGGTCCTTCCTCTTCTCC -3′	5′- AACTTCCCCGTGTTCTATTTGA -3′
**PDE9A**	5′- GTGGGTGGACTGTTTACTGGA -3′	5′- TCGCTTTGGTCACTTTGTCTC -3′
**PDE10A**	5′- GACTTGATTGGCATCCTTGAA -3′	5′- CCTGGTGTATTGCTACGGAAG -3′
**PDE11A**	5′- CCCAGGCGATAAATAAGGTTC -3′	5′- TGCCACAGAATGGAAGATACA -3′
**PBGD**	5′- CAAGGTTTTCAGCATCGCTAC -3′	5′- ATGTCCGGTAACGGCGGC -3′

### Immunohistochemistry

Three-µm lung sections were cut from lung blocks fixed in 3% paraformaldehyde solution. After deparaffinization in xylene and rehydration in a series of grade-decreasing ethanol solutions, the slides were washed in phosphate-buffered saline (PBS). For PDE10A, antigen retrieval was achieved using 0.25% trypsin for 15 min at 37°C; and for proliferating cell nuclear antigen (PCNA), antigen retrieval was achieved by cooking in citrate buffer (pH 6.0) buffer followed by a 15-min incubation in proteinase K at room temperature. The sections were treated with 3% hydrogen peroxide to block endogenous peroxidases prior to serum blocking. Then, a NovaRED horseradish peroxidase (HRP) kit (Vector) was used for PDE10A staining, and a ZymedChem-plus alkaline phosphatase (AP) Polymer kit (Zymed) was used for PCNA staining according to the manufacturer's instructions. The sections were incubated with anti-PDE10A polyclonal antibody (1∶200 in 10% bovine serum albumin, Scottish Biomedical) or anti-PCNA polyclonal antibody (1∶100 in 10% bovine serum albumin) at 4°C overnight. After being washed, slidses were incubated with the corresponding secondary antibody conjugated with HRP or AP for 1 h. After slides were washed, color development was performed with a substrate/chromogen mixture followed by counterstaining with hematoxylin. The sections were examined under a Leica DM 2500 microscope using Leica QWin imaging software (Leica). Sections from 4–5 rats in each group were stained with each antibody. PCNA positive pulmonary vascular cells were counted throughout the entire section and expressed as fold changes of the control lung by calculating the number of PCNA-positive cells per pulmonary vessel [20].

### Immunocytochemistry

Rat PASMCs grown on 8-well chamber slides were fixed with −20°C-cooled acetone-methanol mix (1∶1) for 10 min at 4°C. After being washed 3 times with PBS, the fixed cells were sequentially incubated with blocking buffer (3% bovine serum albumin in PBS) for 1 h at room temperature, primary antibody against PDE10A (1∶200 in blocking buffer, Novus) overnight at 4°C, and FITC-conjugated anti-rabbit secondary antibody (Alexa Fluor® 488 1∶1000 in blocking buffer, Invitrogen) for 1 h at room temperature. Cells were counterstained for nuclei with DAPI and visualized using a Leica DMLA fluorescence microscope and Leica QWin imaging software (Leica). PASMCs from 3 individual rats of each group were stained.

### Immunoblotting

Protein extracted with RIPA buffer (Santa Cruz) was resolved with SDS-PAGE (10% acrylamide) and transferred onto nitrocellulose membranes. After being blocked with 5% non-fat milk for 1 h at room temperature, membranes were probed with rabbit polyclonal anti-PDE10A antibody (1∶2000, Scottish Biomedical), rabbit polyclonal anti-CREB antibody (1∶1000, Millipore), rabbit polyclonal anti-phospho-CREB (Ser133) antibody (1∶1000, Millipore) or mouse monoclonal anti-GAPDH antibody (1∶5000, Novus) overnight at 4°C. Following washing with TBS containing 0.1% Triton X-100, HRP-conjugated secondary antibodies (1∶50000; anti-rabbit, Pierce; anti-mouse, Sigma-Aldrich) were applied for 1 h. After washing, the blots were developed with an enhanced chemiluminescence (ECL) kit (Amersham) followed by film exposure. Blots were repeated three times independently.

### PDE activity assay

3-Isobutyl-1-methylxanthine (IBMX), erythro-9-(2-hydroxy-3-nonyl) adenine (EHNA), rolipram and papaverine were purchased from Sigma-Aldrich. 8MM-IBMX and milrinone were purchased from Calbiochem. Phosphodiesterase enzyme activity was measured using modifications of the methods of Thompson and Appleman [21] and Bauer and Schwabe [22]. Protein was extracted from PASMCs with RIPA buffer (Santa Cruz) and normalized to the same concentration for use. The reactions were performed with 10 µg of protein in 100 µl of HEPES buffer (40 mM; pH 7.6), which consisted of MgCl_2_ (5 mM), bovine serum albumin (1 mg/ml), cAMP (1 µM) and [^3^H]-cAMP (1 µCi/ml, Amersham), at 37°C for 15 min. The samples were boiled for 3 min, subsequently cooled for 5 min and incubated with 25 µl of *Crotalus atrox* snake venom (20 mg/ml, Sigma-Aldrich) for 15 min at 37°C. After being chilled on ice, the samples were applied to QAE Sephadex A-25 (Amersham) mini-chromatography columns and eluted with 1 ml of ammonium formate (30 mM, pH 7.5). The eluents were collected in 2 ml of scintillation solution (Roth), and counts per minute (CPM) were measured by a beta-counter. Each assay was performed in triplicate and repeated twice independently. Data are expressed as pmol cAMP/min/mg protein.

### cAMP enzyme immunoassay (EIA)

At the end of culture, cells were washed twice with PBS and lysed in 0.1 M HCl at room temperature for 10 min. After centrifugation, the supernatants were normalized to the same protein concentration for use. Fifty-µl of protein samples, which were pre-diluted to 0.3 µg/µl, and standard solutions were incubated with 50 µl of tracer and 50 µl of antibody in the dark at 4°C overnight. After washing 5 times, plates were incubated with Ellman's solution for 90–120 min at room temperature with gentle shaking. The plates were read at a wavelength of 405 nm, and the concentration was calculated by the ready-made Cayman EIA Double workbook. The standard curve was generated as a plot of the percent bound/maximum bound (%B/B0) vs concentration of a series of known standards using linear (y) and log (x) axes. Using the 4-parameter logistic equation obtained from the standard curve, the cAMP concentrations of samples were determined, which are given as nmol/mg protein. Each sample was determined in duplicate and repeated twice.

### Proliferation assay

PASMC proliferation was evaluated using the [^3^H]-thymidine incorporation assay. PASMCs (1×10^4^ cells/well) were seeded on 48-well plates and grown overnight. The next day, the medium was substituted with DMEM/F12 containing 0.1% FBS with or without siRNA to render the cells quiescent. After 24-h serum starvation, cells were induced to reenter the cell cycle by 10% FBS, and the cells were incubated with or without papaverine for 24 h. The final 4 h of the incubation included the incorporation of [^3^H]thymidine (0.4 µCi/ml, Amersham). Cells were then washed twice with 500 µl of chilled HBSS, fixed with 250 µl of ice-cold methanol and precipitated by 250 µl of 10% trichloroacetic acid. Finally, samples were lysed in 0.1 M NaOH, transferred to 4 ml of scintillation solution (Roth) and counted by a beta-counter to determine CPM values. All labeling was performed on quadruplicate cultures and repeated twice independently. The proliferation of PASMCs is shown as a percentage (taking the CPM of unstimulated PASMCs under 0.1% FCS as 100%).

### RNA interference

siRNA oligonucleotides specific for PDE10A (sense: 5′-GGACAGCUUGGAUUCUACA-3′; anti-sense: 5′-UGUAGAAUCCAAGCUGUCC-3′) and scramble siRNA (dTdT 3′ overhang) were purchased from Eurogentec and transient transfection of siRNA was performed with X-tremeGENE siRNA Transfection Reagent (Roche) according to the manufacturer's protocols. PASMCs were subcultured to 40% confluence in antibiotic-free DMEM supplemented with 10% FBS and 2 mM L-glutamine. Transfection of 100 nM siRNA (ratio of siRNA to transfection reagent, 1 µg/4 µl) was performed in Opti-MEM (Gibco) for 5 h, which was followed by culturing in DMEM supplemented with 10% FBS and 2 mM L-glutamine for up to 24 h (RNA isolation) or 48 h (protein isolation and enzyme immunoassay, respectively). The RNA interference was well established and repeated three times.

### Statistical analysis

Data are expressed as the mean and standard error of the mean (SEM). All statistical analyses were performed with Student's *t* test for comparisons between two groups or with one-way ANOVA and Newman-Keuls post-hoc test for multiple comparisons. Differences between groups were considered significant at P<0.05.

## Results

### Expression of PDE7-11 in rat lung tissues and PASMCs

**Figure 1:**
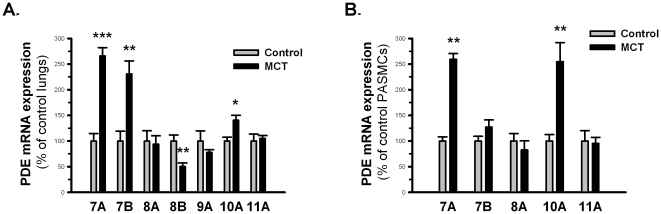
Expression of PDE7-PDE11 isoforms in rat lung tissue and rat PASMCs. **A**) mRNA expression of PDE7-PDE11 in lung homogenates from control rats (gray bars) and MCT-PH rats (black bars) as shown by qRT-PCR after normalization to PBGD. *P<0.05, **P<0.01, ***P<0.001 vs control lungs. n = 4 in each group. **B**) Relative mRNA levels of PDE7-PDE11 in control (gray bars) and MCT (black bars) PASMCs demonstrated by qRT-PCR after normalization to PBGD. **P<0.01 vs control PASMCs. n = 4 in each group. Values are expressed as the mean ± SEM.

The expression of PDE7-11 was investigated by qRT-PCR. In the lungs of MCT-treated rats, we observed upregulation of PDE7A, PDE7B, and PDE10A and downregulation of PDE8B. The other PDEs were expressed at similar levels as the controls (Figure 1A). In the isolated PASMCs at passage 2, we only observed the presence of PDE7A, PDE7B, PDE8A, PDE10A and PDE11A, and we found a 2.5-fold increase in PDE7A and PDE10A mRNA expression in MCT PASMCs compared with control PASMCs (Figure 1B). However, the translational regulation of PDE7A in lung vasculature is beyond the scope of our study due to the fact that PDE7A protein signal was not detectable with the antibodies we possess (data not shown); therefore, we focused on PDE10A.

### PDE10A is selectively upregulated in pulmonary vasculature and in PASMCs from MCT-PH rats

**Figure 2:**
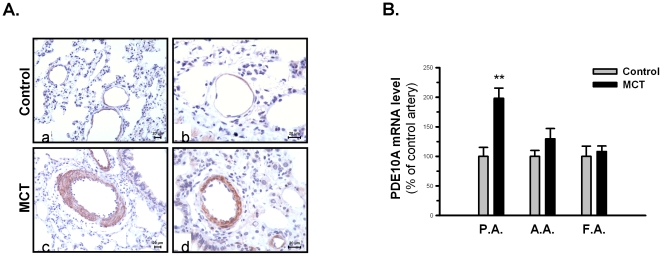
PDE10A expression and localization in rat pulmonary vasculature. **A**) Immunohistochemistry staining of PDE10A in lung sections from control (a, b) and MCT (c, d) rats. Scale bar: 20 µm. **B**) PDE10A mRNA expression in the pulmonary artery (P.A.), aortic artery (A.A.) and femoral artery (F.A.) from control rats (gray bars) and 4-week MCT-PH rats (black bars) are shown as a percentage of control by qRT-PCR after normalization to PBGD. **P<0.01 vs control PASMCs. n = 4 in each group. Values are expressed as the mean ± SEM.

**Figure 3:**
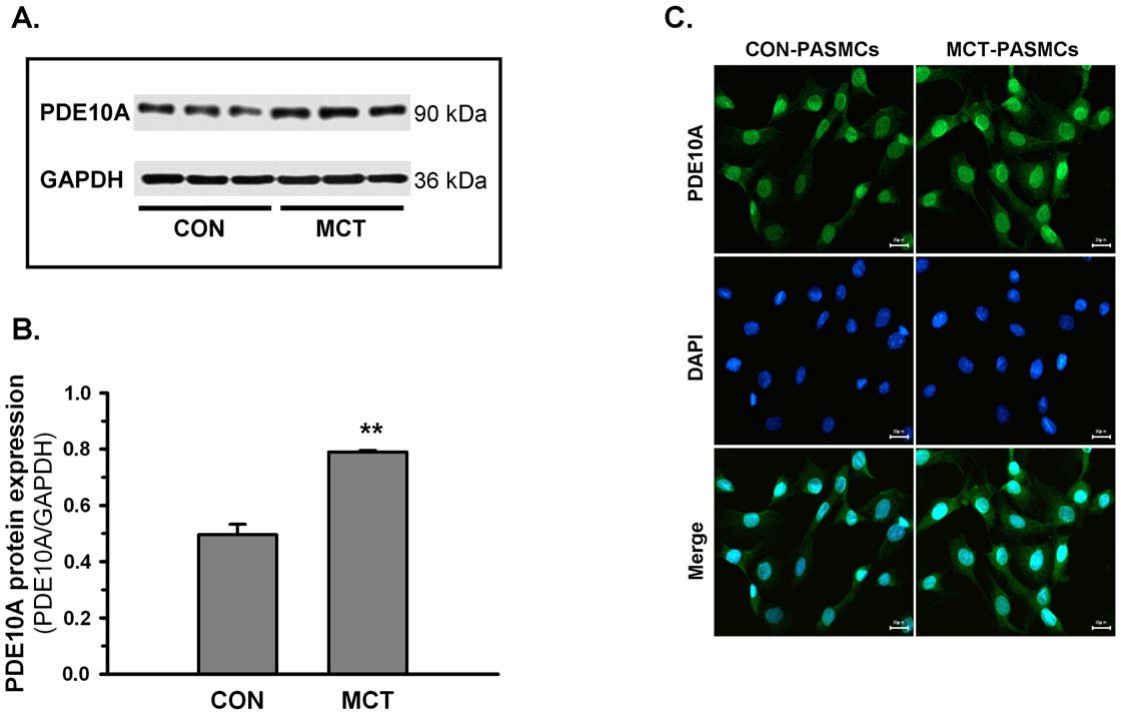
PDE10A expression and localization in PASMCs. **A**) A representative blot of PDE10A protein expression in MCT and control PASMCs, which was determined by immunoblots using GAPDH as the loading control. **B**) Densitometric quantification of PDE10A expression in PASMCs is shown as the ratio to GAPDH in the bar graph. *P<0.05 vs control PASMCs, n = 3 in each group. Values are expressed as the mean ± SEM. **C**) Cellular localization of PDE10A in both control and MCT PASMCs was determined by representative immunofluorescence: green (PDE10A, FITC-conjugated), Blue (DAPI). Staining is shown at 400× magnification.

Because the present study was the first to report PDE10A expression in the lung, immunohistochemistry was performed to verify the PDE10A expression pattern. Figure 2A shows that a stronger immunoreactivity of PDE10A was observed in lung specimens from MCT-PH rats, which suggests that the site-specific change of PDE10A expression was induced in pulmonary hypertensive lungs, especially in the medial layers of pulmonary arteries. In contrast, only weak expression of PDE10A was detected in pulmonary vessels of control rat lungs. In addition, immunoreactivity against PDE10A was also noted in bronchial SMCs in the small airways. To investigate whether PDE10A induction is specific in remodeled pulmonary vasculature, we examined PDE10A expression in pulmonary and systemic arteries, including the aortic and femoral artery. qRT-PCR data showed a 2-fold increase in PDE10A mRNA expression in the pulmonary arteries of MCT-PH rats compared to control rats, which showed no changes in either the aortic artery or the femoral artery (Figure 2B). In corroboration, immunoblotting demonstrated a significant increase in PDE10A expression in MCT PASMCs compared with control (Figure 3A and 3B). In addition, immunofluorescence staining showed a predominant presence of PDE10A in the nuclei of PASMCs (Figure 3C). Similar localization was observed with two additional anti-PDE10A antibodies (data not shown).

### Higher contribution of PDE10A to the total cAMP PDE activity in MCT PASMCs compared with control PASMCs

**Figure 4:**
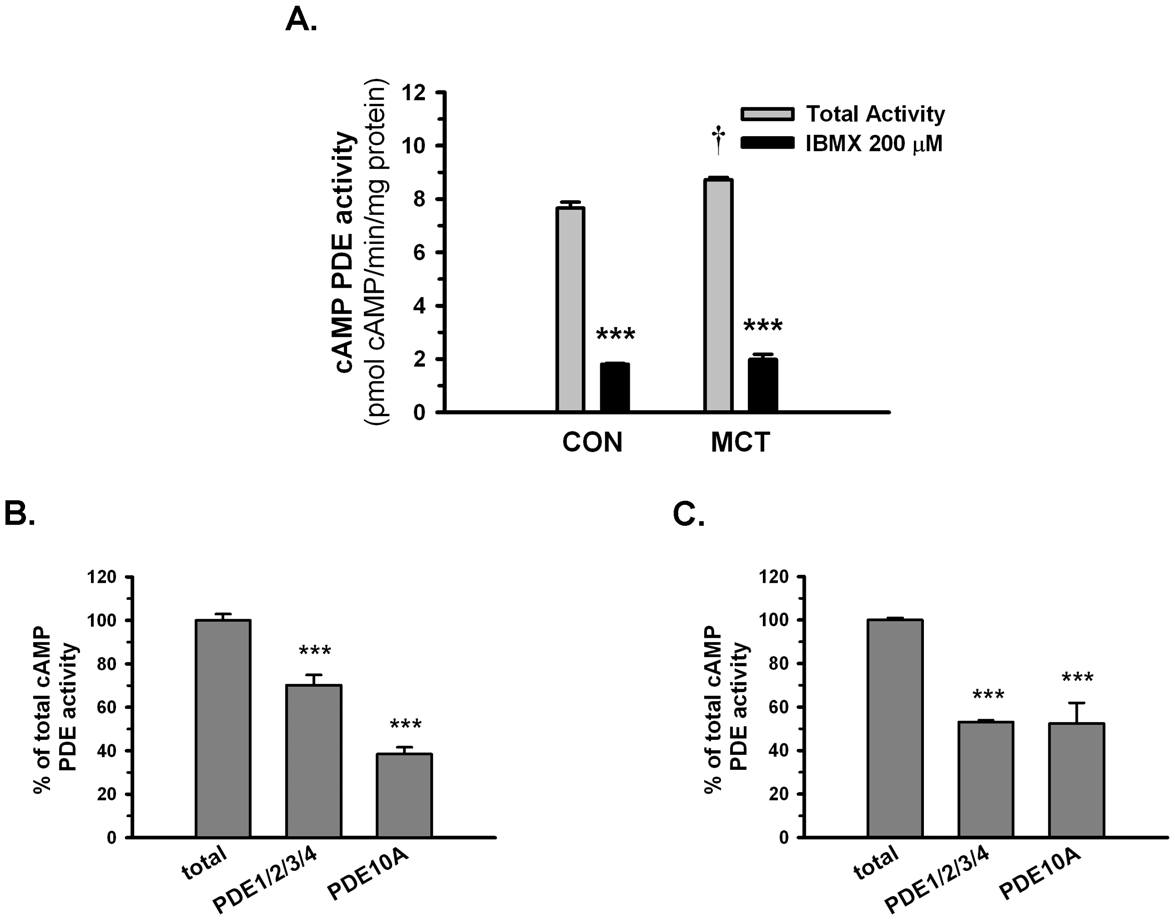
cAMP PDE activities of control and MCT PASMCs. **A**) Total cAMP activity in control and MCT PASMCs. †P<0.05 vs control PASMCs, ***P<0.001 vs total activity, n = 3 in each group. cAMP activity of **B**) control PASMCs and **C**) MCT PASMCs mediated by PDE10A or other PDEs (PDE1, PDE2, PDE3 and PDE4). Total cAMP PDE activity was suppressed in varying degrees by a PDE10A inhibitor (papaverine, 10 µM) or by a combination of inhibitors against PDE1 (8 mm-IBMX, 30 µM), PDE2 (EHNA, 30 µM), PDE3 (milrinone, 5 µM) and PDE4 (rolipram, 10 µM). ***P<0.001 vs total activity, n = 3 in each group. Values are expressed as the mean ± SEM.

In addition to the expression level, the enzyme activity of PDE10A was also determined by the PDE activity assay. The total cAMP hydrolyzing PDE activity was increased in MCT PASMCs compared to control PASMCs (8.72 vs 7.66 pmol cAMP/min/mg protein), and this activity was suppressed by a non-selective PDE inhibitor, IBMX, to a similar basal level (1.9 pmol cAMP/min/mg protein) (Figure 4A). Interestingly, the PDE10A accounted for 53% of the total cAMP PDE activity in MCT PASMCs as opposed to 38% in control PASMCs (Figure 4B and 4C). In contrast, the contribution of other cAMP hydrolyzing PDEs (PDE1, PDE2, PDE3 and PDE4) declined from 70% in control PASMCs to 52% in MCT PASMCs. Taken together, these data suggest that PDE10A is one of the major cAMP hydrolyzing PDEs in PASMCs, and the cAMP hydrolyzing activity contributed by PDE10A was significantly increased in PASMCs from MCT-PH rats.

### PDE10A knockdown by siRNA inhibits PASMC proliferation

**Figure 5:**
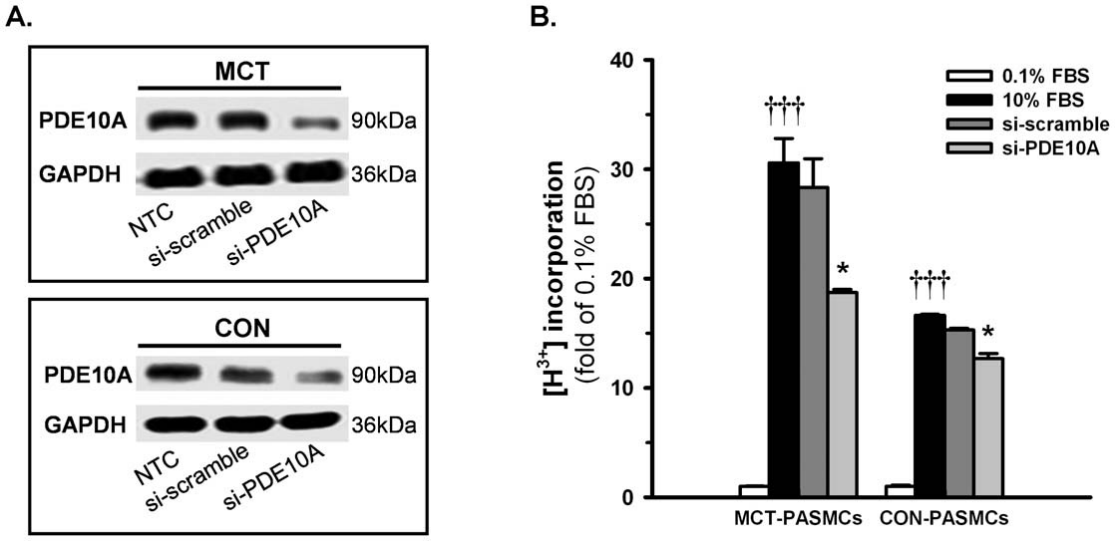
Effects of PDE10A knockdown by siRNA on PASMC proliferation. Representative blots showing PDE10A protein expression in **A**) control PASMCs and **B**) MCT PASMCs transiently transfected with 100 nM scramble siRNA or PDE10A siRNA for 48 h. PASMCs treated with transfection reagent alone served as a negative control (NTC), and GAPDH was used as a loading control. **C**) PASMC proliferation stimulated by 10% FBS with siRNA transfection was determined by the [^3^H]thymidine incorporation assay. †††P<0.001 vs 0.1% FBS, *P<0.05 vs 10% FBS, n = 4 in each group. Values are expressed as the mean ± SEM.

The immunoblotting results in Figure 5A show that endogenous PDE10A protein expression in control and MCT PASMCs was strongly suppressed by PDE10A siRNA (100 nM), whereas no change occurred after transfection with scramble siRNA. In addition, to examine the isoform-specific effects of PDE10A siRNA, the expression of other cAMP-PDE isoforms was analyzed after PDE10A siRNA transfection. Quantitative RT-PCR results suggest that PDE10A siRNA suppressed PDE10A mRNA expression by 75% without affecting PDE1A, PDE3A, PDE3B and PDE4A expression (Figure S1). [^3^H]-thymidine incorporation stimulated by 10% FBS indicated that MCT PASMCs were 80% more proliferative than control PASMCs (Figure 5B). Furthermore, the proliferation of MCT PASMCs was reduced by 40% of the original level after PDE10A knockdown, whereas proliferation was only reduced by 25% of the original level in control PASMCs (Figure 5B).

### Pharmacological inhibition of PDE10A *in vitro* suppresses PASMC proliferation, accumulates intracellular cAMP and activates cAMP response element binding protein (CREB)

**Figure 6:**
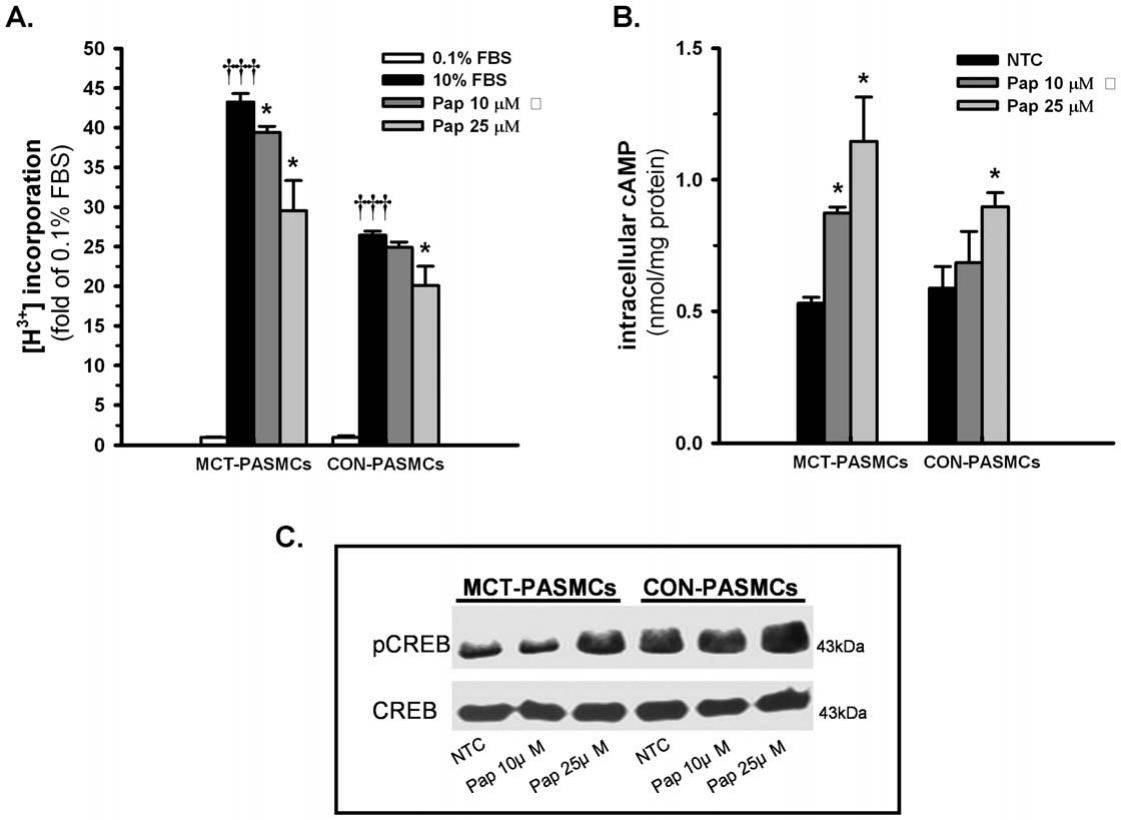
Effects of the PDE10A inhibitor papaverine (Pap) on PASMC proliferation, intracellular cAMP accumulation and CREB activation. **A**) FBS stimulated PASMC proliferation after Pap (10 µM, 25 µM) treatment for 24 h. [^3^H]thymidine incorporation was used to evaluate cell proliferation. †††P<0.001 vs 0.1% FBS, *P<0.05 vs 10% FBS, n = 4 in each group. **B**) Intracellular cAMP levels in PASMCs after Pap treatment measured by a cAMP enzyme immunoassay. The cAMP content of PASMC lysates is given as nmol/mg protein. *P<0.05, ***P<0.001 vs NTC (negative control), n = 4 in each group. **C**) CREB phosphorylation after Pap (10 µM, 25 µM) treatment. Representative immunoblots are shown in the figure, Values are expressed as the mean ± SEM.

Administration of the PDE10 inhibitor papaverine (Pap, 25 µM) resulted in a 40% reduction in [^3^H]-thymidine incorporation in MCT PASMCs compared to a 25% reduction in control PASMCs (Figure 6A). The cAMP EIA assay data suggested that intracellular cAMP levels were increased 2.1-fold in MCT PASMCs after Pap (25 µM) treatment (Figure 6B), whereas there was only a 1.5-fold increase in cAMP levels in control PASMCs. Because CREB is an important downstream target of cAMP, we investigated whether CREB was activated by increased cAMP levels following PDE10 inhibition by determining the phosphorylation of serine 133 (Ser133) via immunoblotting. Figure 6C shows that control PASMCs exhibited high levels of CREB phosphorylation, which increased slightly after PDE10A inhibition by Pap (25 µM). In contrast, Pap (25 µM) dramatically increased the phosphorylation of CREB (Ser33) in MCT PASMCs.

### Therapeutic effects of papaverine in MCT-PH rats *in vivo*

**Figure 7:**
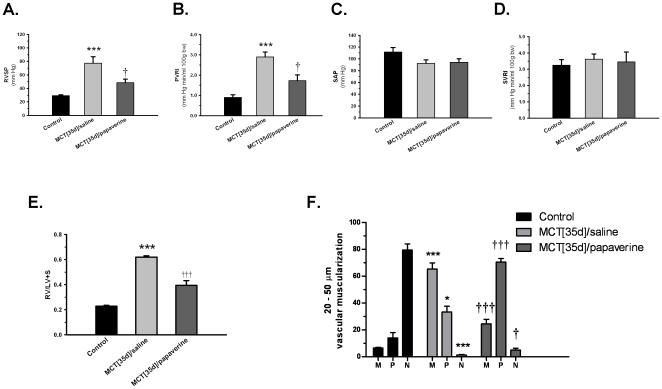
Effects of papaverine on MCT-PH rats *in vivo*. Papaverine was applied by continuous intravenous infusion with osmotic minipumps from days 21 to 35. A) RVSP (mm Hg), B) SAP (mm Hg), C) PVRI (mm Hg min/ml 100 g body), and D) SVRI (mm Hg min/ml 100 g body) are given as the mean ± SEM. ***P<0.001 vs control; †P<0.05 vs. MCT [35 d]/saline. Control: n = 9; MCT [35 d]/saline: n = 8; MCT [35 d]/papaverine: n = 8. E) Right heart mass, which was measured by the ratio of RV/LV+S. F) Effect of papaverine on the extent of muscularization of peripheral pulmonary arteries. The percentage of nonmuscularized (N), partially muscularized (P), or fully (M) muscularized pulmonary arteries related to the total number of pulmonary arteries is given as the mean ± SEM. A total of 80 to 100 intra-acinar vessels were analyzed in the lung of each rat from each group. ***P<0.001 vs control; †P<0.05 vs MCT [35 d]/saline. Values are expressed as the mean ± SEM.

We performed *in vivo* experiments with an MCT-PH rat model to examine the therapeutic efficacy and anti-remodeling potential of PDE10 inhibition. Compared with control rats, saline treated MCT-PH rats exhibited significant increases in right ventricular systolic pressure (RVSP) on day 35 (77.2±9.7 vs 29.1±1.3 mm Hg) (Figure 7A) and in the pulmonary vascular resistance index (PVRI) (2.90±0.24 vs 0.90±0.13 mm Hg min/ml 100 g body weight) (Figure 7B). We did not observe any significant changes in systemic arterial pressure (SAP) or the systemic vascular resistance index (SVRI) (Figure 7C and 7D). Continuous infusion of papaverine from day 21 to day 35 resulted in a significant reduction of RVSP (48.4±5.3 mm Hg) (Figure 7A) and PVRI (1.73±0.28 mm Hg min/ml 100 g body weight) (Figure 7B), whereas SAP and SVRI did not change significantly (Figure 7C and 7D) compared with saline treated MCT-PH rats. In addition, the ratio of right ventricle (RV)/left ventricle plus septum (LV+S) increased from 0.23±0.01 in control rats to 0.62±0.01 in MCT-PH rats due to increased pulmonary arterial pressure, whereas papaverine treatment significantly reduced this ratio to 0.39±0.04 (Figure 7E).

**Figure 8:**
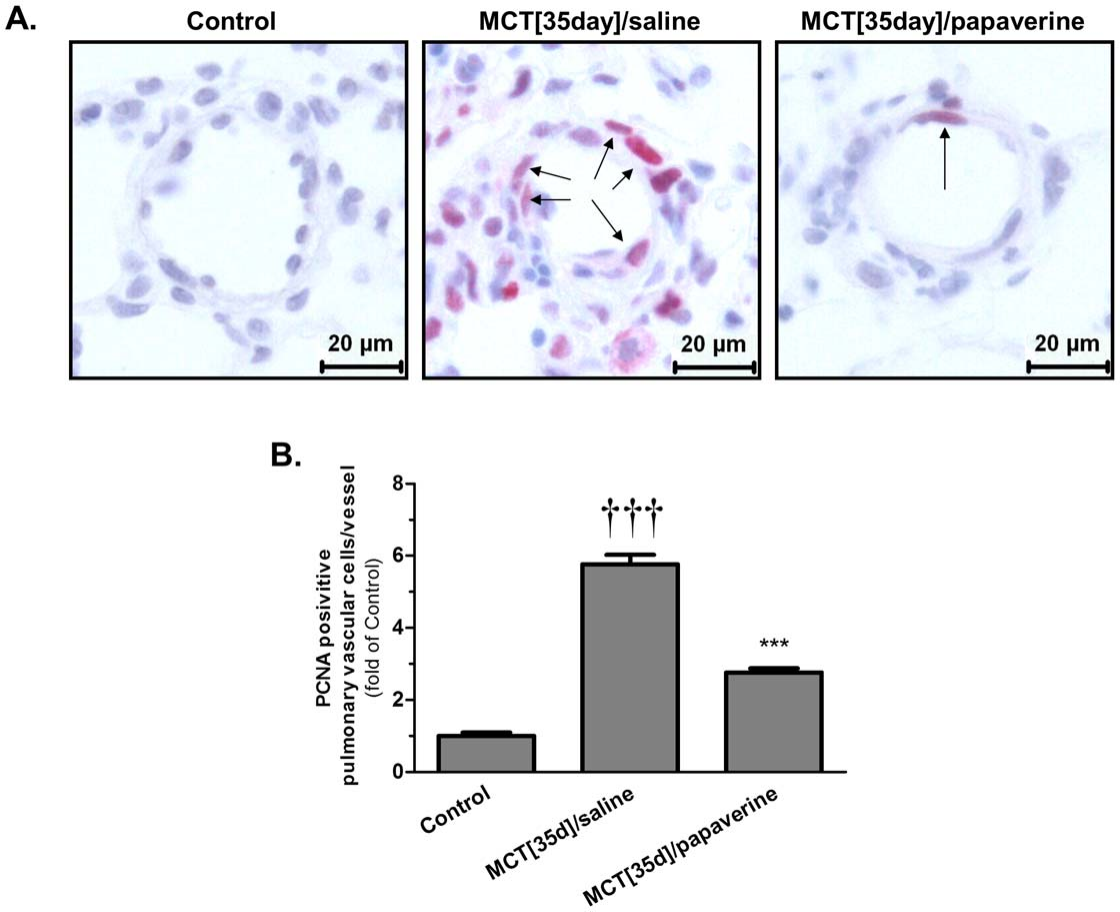
Anti-proliferative effects of papaverine *in vivo*. Proliferating cell nuclear antigen (PCNA) staining was performed to identify proliferating pulmonary vascular cells. **A**) Representative PCNA immunostaining microphotographs of the rat lung sections from control (left), MCT [35 day]/saline placebo (middle) and MCT [35 day]/papaverine (right), with black arrows indicating the PCNA-positive vascular cells in red. Scale bar: 20 µm. **B**) Effects of papaverine on pulmonary vascular cell proliferation are expressed as fold changes compared to control lungs, n = 5 in each group. ***P<0.001 vs control; †††P<0.001 vs MCT [35 d]/saline. Values are expressed as the mean ± SEM.

Quantitative morphological analysis of the degree of muscularization of the peripheral small pulmonary arteries showed that control rat lungs primarily consisted of nonmuscularized arteries (79.5±4.5%) with a small percentage of partially muscularized arteries (14.0±4.0%) and a smaller percentage of fully muscularized arteries (6.5±0.5%) (Figure 7F and Figure S2). In contrast, the percentage of nonmuscularized arteries in saline-treated MCT-PH rat lungs was 1.2±0.4%, and the percentages of partially and nonmuscularized arteries were 33.3±4.3% and 65.4±4.5%, respectively. Papaverine treatment decreased the proportion of fully muscularized arteries to 24.4±3.4%, which was accompanied by significant increases in the proportions of partially muscularized arteries and nonmuscularized pulmonary arteries (70.5±2.7% and 5.0±1.2%, respectively). In addition, compared to the vessels of the control lungs, PCNA staining indicated a 5.8-fold increase in the number of proliferative vascular cells in MCT-PH rat lungs. Importantly, papaverine-treated lungs only had a 2.8 fold increase in the number of proliferative vascular cells, which suggested an amelioration of *in vivo* proliferation by papaverine (Figure 8).

### PDE10A expression in human lungs from donors and idiopathic PAH (IPAH) patients

**Figure 9:**
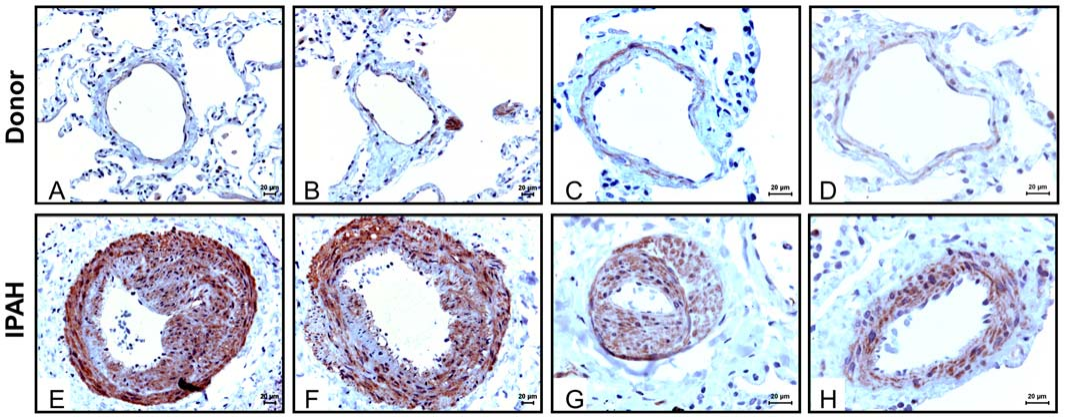
Pulmonary vascular expression and localization of PDE10A in lung tissues from donor and IPAH patients. Representative PDE10A immunostaining microphotographs of the human lung sections from donors (A–D) and IPAH patients (E–H). Scale bar: 20 µm.

To ascertain the clinical relevance of our findings in the MCT-PH rat model, the expression and localization of PDE10A were investigated in human IPAH lungs by immunohistochemistry. As shown in Figure 9, strong immunoreactivity of PDE10A was observed in pulmonary arteries, predominantly in the medial layer of IPAH lung tissue (Figure 9E–H). In contrast, only weak expression of PDE10A was detected in the pulmonary arteries of donor lung tissue (Figure 9A–D).

## Discussion

The present data demonstrate that PDE10A expression is prominently induced in the structurally remodeled arterial muscular layer in pulmonary hypertensive lungs, which suggests that PDE10A contributes to the pathogenesis of pulmonary vascular remodeling. *In vitro* functional deletion of PDE10A in PASMCs resulted in increased cAMP generation, increased CREB phosphorylation and decreased proliferation. *In vivo* PDE10A inhibition by intravenous infusion of papaverine resulted in 40%–50% inhibition of MCT-induced hemodynamic parameters and histological changes. This study supports a central role of PDE10A in progressive pulmonary vascular remodeling and suggests that inhibition of PDE10A is a novel therapeutic approach against pulmonary vascular remodeling for the treatment of PAH.

PDE10A is one of the most recently described PDEs, and it was characterized as a dual-substrate gene in 1999 in mouse and in human brain [23]–[25]. PDE10A has the capacity to hydrolyze both cAMP and cGMP; however, the K_m_ for cAMP is approximately 0.05 µM compared with 3 µM for cGMP. Interestingly, the V_max_ for cAMP hydrolysis is fivefold lower than that for cGMP. Because of this kinetic pattern, PDE10A-mediated cGMP hydrolysis is potently inhibited by cAMP *in vitro*, which suggests that PDE10A may function as a cAMP-PDE and a cAMP-inhibited cGMP-PDE *in vivo* [23]–[25]. Furthermore, the cGMP-binding regulatory (GAF) domain of PDE10A is unique compared to the GAF domains of other PDE families because it is the only one that binds to cAMP instead of cGMP, which may contribute to the preferential hydrolysis of Camp [26]. The unique distribution of PDE10A in the brain and its enrichment in the striatum indicate that PDE10A inhibitors are potential therapeutic agents for the treatment of neurological and psychiatric disorders [27]. Recently, PDE10A-selective inhibitors have also been suggested to be useful in the treatment of diabetes and obesity [24], [28]. In the present studies, we observed a higher expression of PDE10A in the MCT-PH rat pulmonary arteries and isolated PASMCs, which suggests that PDE10A may contribute to the proliferative phenotype of PASMCs. Although the specific functional role of PDE10A in lung tissue needs to be characterized in more detail, the present study also suggests a reactivation of PDE10A signaling in abnormal proliferative lung disease tissues, such as the tissues observed in pathological vascular remodeling. Moreover, PDE10A immunoreactivity was markedly increased in pulmonary arteries of IPAH patient lungs compared to the donor lungs, which indicates the clinical relevance of the findings obtained from the MCT-PH rat model.

Vascular remodeling, which includes the proliferation and hypertrophy of SMCs, is a characteristic feature of PAH. Recent studies have reported that targeting abnormal PASMC proliferation in the vascular media blocks the development of PAH and attenuates pulmonary arterial remodeling in rodents and humans [17], [29]. In our studies, downregulation of PDE10A by siRNA led to a greater suppression of PASMC proliferation among hyperproliferative cells than among healthy control cells, which suggests that PDE10A could be a useful therapeutic target for pulmonary vascular remodeling and other disorders characterized by increased PASMC proliferation. Furthermore, applying the selective PDE10A inhibitor papaverine also suppressed the proliferation of MCT PASMCs to a greater extent than control PASMCs. These anti-proliferative effects are largely due to an increase in intracellular cAMP levels that may stimulate the activity of protein kinase A (PKA) [30], [31]. In line with this notion, several studies from our group and others have reported that compounds that activate adenylate cyclase or inhibit PDE counteract several pathways involved in SMC proliferation [11], [15], [32]. For example, cAMP in vascular SMCs was shown to decrease the expression of cyclin D1 and cdk2 as well as the activation of extracellular signal-regulated kinase (ERK). In addition, cAMP in vascular SMCs has been shown to increase the expression of the anti-proliferative molecules p53 and p21 [33]–[35]. Furthermore, there is now substantial evidence that cAMP/PKA signaling acts as a molecular gate to block cell cycle progression, primarily via occupancy of the cAMP response elements (CREs) in the promoter region of the cyclin A gene and increases in the level and phosphorylation status of the transcription factor CREB [36]. In accordance, we found that CREB phosphorylation was markedly increased in MCT PASMCs after PDE10A inhibition by papaverine. We only observed a minor increase in control PASMCs, which suggested that hyperproliferative PASMCs were more sensitive to PDE10A inhibition. This may have important consequences for pulmonary media wall remodeling in PAH *in vivo* because CREB content has been shown to be diminished in smooth muscle cells in remodeled pulmonary arteries with PAH [37].

More importantly, numerous studies have demonstrated that the compartmentalization and dynamics of cAMP signaling are crucial for cAMP-triggered cellular responses [38]. In contrast to the conventional transmembrane adenylyl cyclase, soluble adenylyl cyclase (sAC) is distributed in specific subcellular compartments, including mitochondria, centrioles, mitotic spindles, and nuclei. sAC has been proved to be an alternative source of intracellular cAMP pools regulated in a temporary and spatial manner [39]. Furthermore, the direct activation of nuclear PKA by nuclear-localized cAMP was demonstrated to be a more efficient signaling pathway leading to CREB activation compared to activation of cytoplasmic PKA [40]. Notably, the subcellular distribution of PDE proteins may regulate the specific intracellular localization of cAMP by compartmentalized hydrolysis of cAMP [41]. As we showed in the present study, PDE10A was localized in the nuclei of PASMCs with great abundance. Furthermore, PDE10 inhibition led to cAMP accumulation and significant activation of the transcriptional factor CREB, which was followed by cAMP accumulation. PDE10 inhibition may lead to increased cAMP in the nuclei of PASMCs and regulate cell growth responses by activating CREB. Further investigations on the subcellular distribution of cAMP after PDE10 inhibition, however, are required to prove this finding.

Recently, Murray et al. demonstrated that the total PDE activity levels are increased in PAH PASMCs compared to control PASMCs [42]. In agreement with these findings, an increase in the total cAMP-PDE activity was observed in our experimental model of PAH. Notably, the relative contribution of PDE10A to the total cAMP-PDE activity was increased in MCT PASMCs compared to control PASMCs, which indicated that PDE10A inhibition was more effective in increasing cAMP generation and inhibiting the hyperproliferation of PASMCs from MCT-PH rats. This suggests that PDE inhibitors that increase cAMP levels in general, as well as PDE10A-selective inhibitors, offer new possibilities for therapeutic intervention for pulmonary vascular remodeling in PH. In line with these findings, treatment of MCT-PH rats with the PDE10A inhibitor papaverine for 14 days markedly improved their hemodynamics. Indeed, RVSP and PVRI values were significantly lowered after papaverine treatment. The structural changes of the lung vasculature, such as the high percentage of fully muscularized peripheral pulmonary arteries and proliferating vascular cells, were significantly decreased after papaverine treatment. In addition, right heart hypertrophy was significantly reduced by papaverine. Because papaverine is used as a potent vasodilator in the systemic and cerebral vasculature, we could not eliminate the possibility that it may exert vasodilatory effects on pulmonary vessels [43]. Papaverine-induced vasorelaxation is believed to be related to reduced calcium influx following PKA activation after cAMP levels increase [44], [45]. Nevertheless, the present study showed that the systemic pressures of papaverine-treated rats are similar to those of healthy rats. The effects of papaperine may not be exclusively targeted to PDE10A, although a continuous intravenous infusion of papaverine should not result in high plasma levels of this compound. Based on our previous experience in the field of *in vivo* siRNA mediated knockdown, we believe that *in vivo* siRNA targeting of PDE10A in pulmonary arterial SMCs is not feasible because the cells are present between endothelial and fibroblast cells in pulmonary vasculature. Within published literature in the field of PH, only two manuscripts have addressed the siRNA mediated knockdown of endothelial specific genes [46], [47]. Hence, new PDE10 inhibitors with higher selectivity and potency are required to explore these therapeutic aspects in more detail.

In conclusion, PDE10A expression and activity are increased in PASMCs of experimental MCT-PH rats. Using PDE10A-targeted siRNA and the PDE10 inhibitor papaverine, we demonstrated that PDE10A plays a major role in the hyperproliferation of PASMCs. Furthermore, papaverine significantly improved pulmonary hemodynamics and significantly reversed the structural abnormalities underlying the MCT-PH rat model. To the best of our knowledge, this was the first study addressing a central role of PDE10A in progressive pulmonary vascular remodeling, and we propose the inhibition of PDE10A as a novel therapeutic approach to the treatment of PAH.

## Supporting Information

Figure S1 **mRNA expression of cAMP-PDEs (PDE10A, PDE1A, PDE3A, PDE3B and PDE4A) after PDE10A siRNA transfection.** To examine the isoform-specific effects of PDE10A siRNA, cAMP-PDEs were analyzed by qRT-PCR after a 24-h transfection of 100 nM scramble siRNA (gray bars) or PDE10A siRNA (black bars). PASMCs treated with transfection reagent alone were used as a negative control (NTC), n = 3 in each group. Values are expressed as the mean ± SEM.
(TIF)

Figure S2 **Representative double immunostaining microphotographs of the rat lung sections were used to assess the muscularization of small pulmonary arteries.** Staining was undertaken for von Willebrand factor (brown; endothelial cells) and α-smooth muscle actin (purple; smooth muscle cells).
(TIF)
