# Supplementary figures and images for: Phosphodiesterase 10A Upregulation Contributes to Pulmonary Vascular Remodeling
Source: PLoS One. 2011 Apr 11;6(4):e18136. doi: 10.1371/journal.pone.0018136 (PMC3073929; doi:10.1371/journal.pone.0018136)

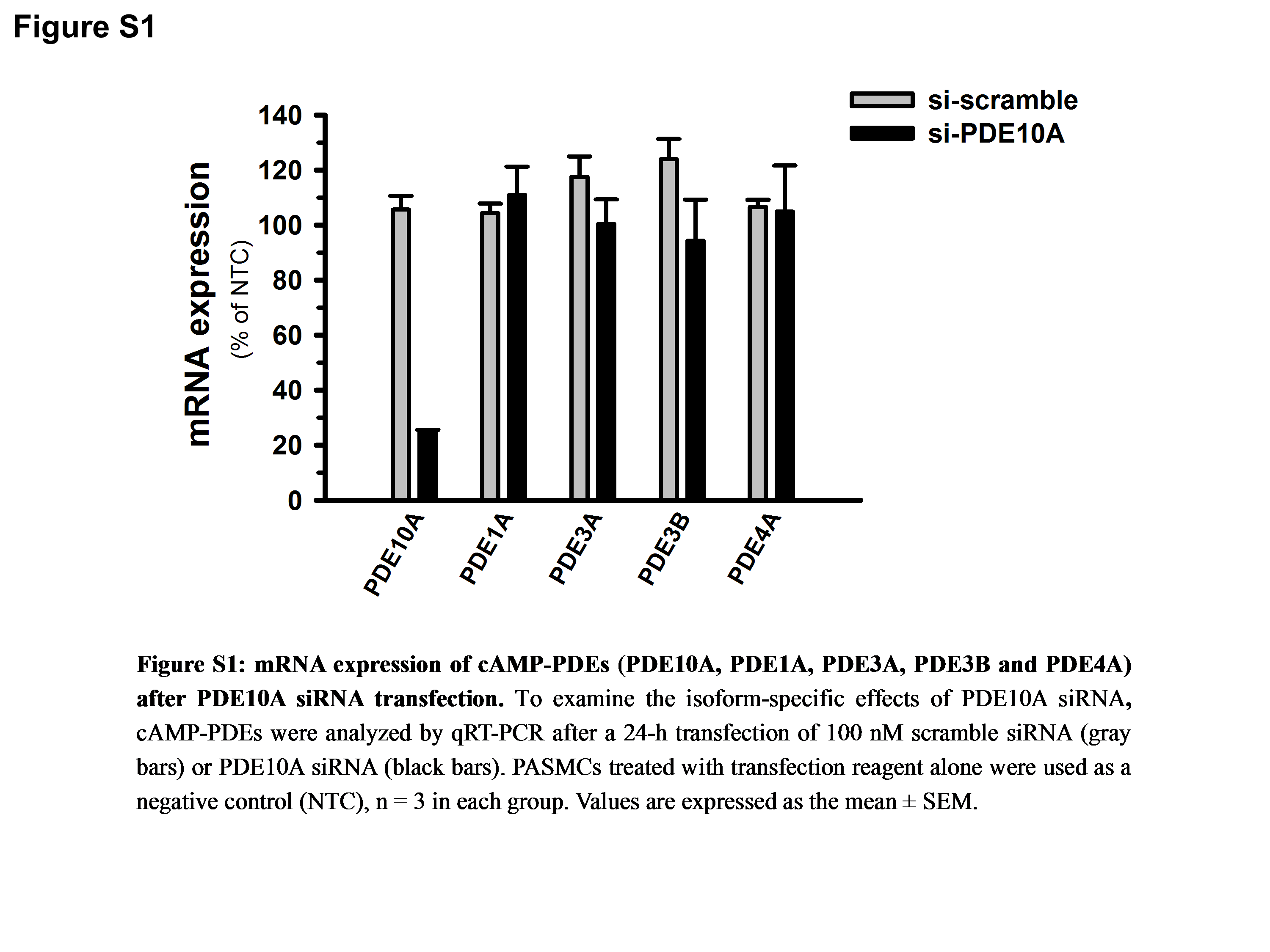

Supplement: Figure S1 — mRNA expression of cAMP-PDEs (PDE10A, PDE1A, PDE3A, PDE3B and PDE4A) after PDE10A siRNA transfection. To examine the isoform-specific effects of PDE10A siRNA, cAMP-PDEs were analyzed by qRT-PCR after a 24-h transfection of 100 nM scramble siRNA (gray bars) or PDE10A siRNA (black bars). PASMCs treated with transfection reagent alone were used as a negative control (NTC), n = 3 in each group. Values are expressed as the mean ± SEM. (TIF) [file pone.0018136.s001.tif]

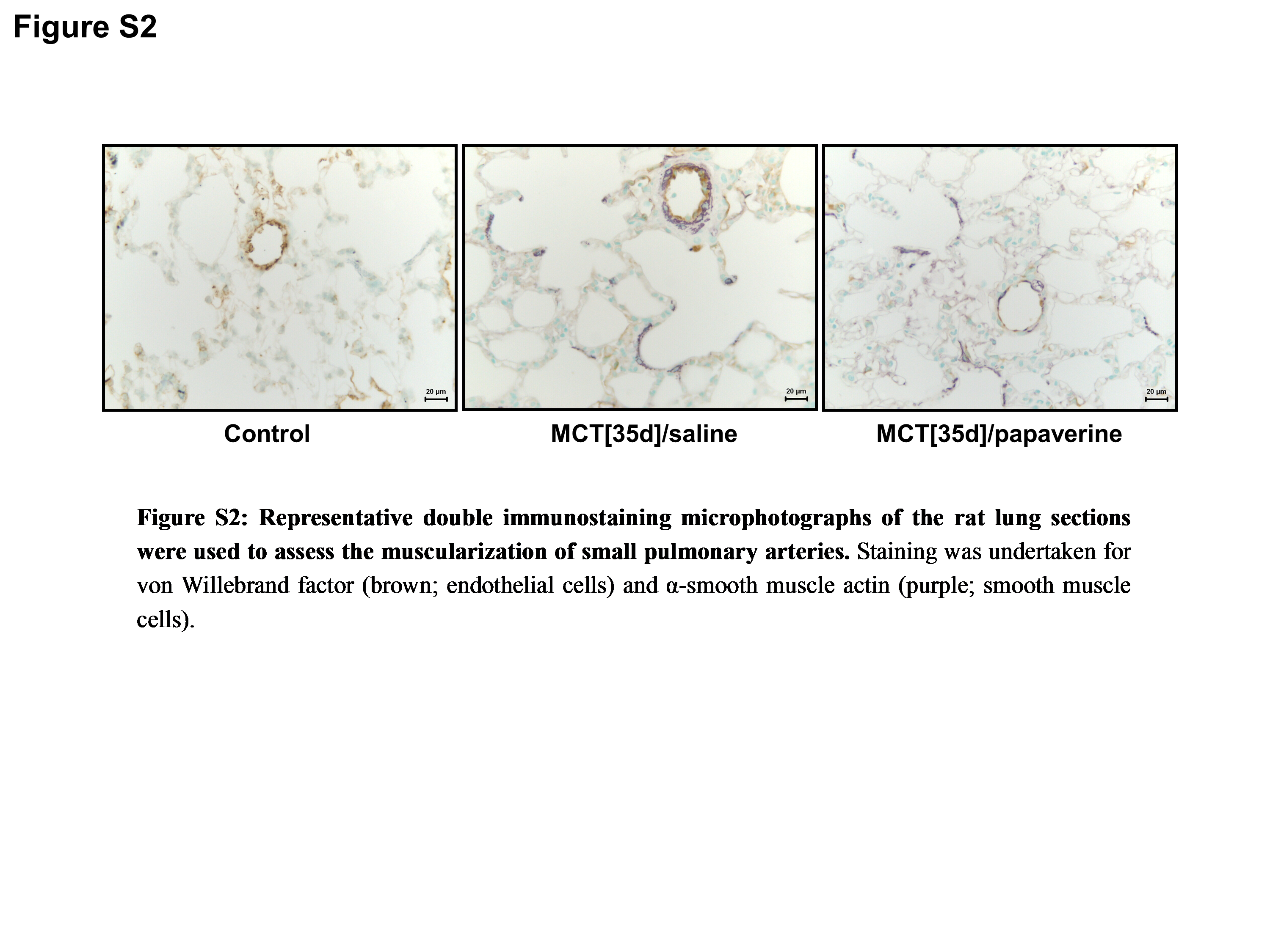

Supplement: Figure S2 — Representative double immunostaining microphotographs of the rat lung sections were used to assess the muscularization of small pulmonary arteries. Staining was undertaken for von Willebrand factor (brown; endothelial cells) and α-smooth muscle actin (purple; smooth muscle cells). (TIF) [file pone.0018136.s002.tif]
